# MicroRNA-Mediated Regulation of the Virus Cycle and Pathogenesis in the SARS-CoV-2 Disease

**DOI:** 10.3390/ijms222413192

**Published:** 2021-12-07

**Authors:** Rosalia Battaglia, Ruben Alonzo, Chiara Pennisi, Angela Caponnetto, Carmen Ferrara, Michele Stella, Cristina Barbagallo, Davide Barbagallo, Marco Ragusa, Michele Purrello, Cinzia Di Pietro

**Affiliations:** Department of Biomedical and Biotechnological Sciences, University of Catania, 95123 Catania, Italy; rosalia.battaglia@unict.it (R.B.); rubensalonzo@gmail.com (R.A.); pennisi.chiara@yahoo.it (C.P.); caponnettoangela@gmail.com (A.C.); carmen-ferrara@live.com (C.F.); michelestella7@gmail.com (M.S.); barbagallocristina@gmail.com (C.B.); dbarbaga@unict.it (D.B.); mragusa@unict.it (M.R.); purrello@unict.it (M.P.)

**Keywords:** SARS-CoV-2, COVID-19, variants of concern (VOCs), human microRNAs, viral microRNAs

## Abstract

In the last few years, microRNA-mediated regulation has been shown to be important in viral infections. In fact, viral microRNAs can alter cell physiology and act on the immune system; moreover, cellular microRNAs can regulate the virus cycle, influencing positively or negatively viral replication. Accordingly, microRNAs can represent diagnostic and prognostic biomarkers of infectious processes and a promising approach for designing targeted therapies. In the past 18 months, the COVID-19 infection from SARS-CoV-2 has engaged many researchers in the search for diagnostic and prognostic markers and the development of therapies. Although some research suggests that the SARS-CoV-2 genome can produce microRNAs and that host microRNAs may be involved in the cellular response to the virus, to date, not enough evidence has been provided. In this paper, using a focused bioinformatic approach exploring the SARS-CoV-2 genome, we propose that SARS-CoV-2 is able to produce microRNAs sharing a strong sequence homology with the human ones and also that human microRNAs may target viral RNA regulating the virus life cycle inside human cells. Interestingly, all viral miRNA sequences and some human miRNA target sites are conserved in more recent SARS-CoV-2 variants of concern (VOCs). Even if experimental evidence will be needed, in silico analysis represents a valuable source of information useful to understand the sophisticated molecular mechanisms of disease and to sustain biomedical applications.

## 1. Introduction

In the 21st century, humanity faced the beginning of a worldwide epidemic outbreak caused by the severe acute respiratory syndrome coronavirus 2 (SARS-CoV-2) [1], a highly transmissible and pathogenic coronavirus that causes an acute respiratory disease, named by the World Health Organization (WHO) “coronavirus disease 2019” (COVID-19). The origin of SARS-CoV-2 infection was first reported in people exposed in a seafood market in Wuhan City, China, in December 2019, and is thought to have passed from bats to humans through a possible intermediary host [2]. Since the initial reports of the first cluster of COVID-19 pneumonia, infections from SARS-CoV-2 have spread worldwide, causing more than about 237 263 million confirmed cases and 5.2 million deaths to date (3 December 2021) (https://www.who.int/emergencies/diseases/novel-coronavirus-2019).

SARS-CoV-2 is an enveloped, positive-sense, single-stranded RNA virus of 29,891 nucleotides that belongs to the order Nidovirales, family Coronaviridae, and subfamily Orthocoronavirinae [3,4]. High-throughput sequencing identified SARS-CoV-2 as a betacoronavirus (β-CoV), which shared about 79% and 50% [5] of its genetic sequence with SARS-CoV-1, associated with the severe acute respiratory syndrome (SARS) that emerged in 2002, and the Middle East respiratory syndrome coronavirus (MERS-CoV), responsible for another atypical respiratory disease that occurred in 2012 [6], respectively. Similar to other βCoVs, the SARS-CoV-2 genome contains two flanking untranslated regions (UTRs) and a single long open reading frame encoding a polyprotein arranged in the order from 5′ to 3′: Replicase (ORF1a/ab), Spike (ORF S), Envelope (ORF E), Membrane (ORF M), and Nucleocapsid (ORF N). There are at least six accessory proteins (3a, 6, 7a, 7b, 8, and 10) typical of SARS-CoV-2 and it lacks the hemagglutinin-esterase gene, which is a characteristic of the β-CoVs lineage [3,7,8]. ORF1a and ORF1ab are translated into two polyproteins (pp), pp1a and pp1ab, and then cleaved by viral proteases into 16 Non-Structural Proteins (NSPs), some of which are essential for virus transcription and replication. NSP12, for example, is an RNA-dependent RNA polymerase (RdRp) that is part of the replication transcription complex (RTC), which reads the viral genome template in the 3′ to 5′ direction [8]. Viral genomic replication begins with the synthesis of full-length negative-sense genomic copies, which function as a template for the generation of new positive-sense genomic RNA used to translate more NSPs and RTCs or to be packaged into new virions [4]. Conversely to the other βCoVs, SARS-CoV-2 is markedly more infectious and has different epidemiological dynamics and pathogenic effects [2,6].

COVID-19 can result in a wide spectrum of clinical manifestations, from asymptomatic or paucisymptomatic infection to acute respiratory distress syndrome and multiple organ failure and death [9]. Severe COVID-19 is accompanied by an inappropriate pro-inflammatory host immune response and a diminished antiviral interferon response [10] that has been associated with severe or moderate disease [11]. Finally, entering through the specific binding of the spike protein to angiotensin-converting enzyme2 receptors, SARS-CoV-2 can damage endothelial cells, leading to inflammation, thrombi, acute lung damage, followed by pulmonary fibrosis and chronic impairment of lung function, and brain damage with long-term neurological sequelae [12,13]. In particular, lung damage represents the principal hurdle to recovery in severe patients [14]. Despite the countless papers published over the last 18 months on SARS-CoV-2, the cellular and molecular mechanisms of its pathogenesis, and above all, the individual response to infection, are not completely understood. Moreover, to date, there are no confirmed therapeutic strategies, and even mass vaccination has yet to be evaluated, especially in response to the virus variants.

Several SARS-CoV-2 variants, which seem to exhibit increased transmissibility, recently emerged following virus genetic diversification, consistent with the adaptation to humans, fueled by its massive circulation worldwide [15].

Based on their epidemiological characteristics and patterns of spike mutations, five variants have been classified as variants of concern (VOCs) by the WHO (3 December 2021) https://www.who.int/en/activities/tracking-SARS-CoV-2-variants/.

They are defined by multiple convergent mutations that are hypothesized to have arisen either in the context of chronic infections or in previously infected individuals [16,17].

B.1.1.7, B.1.351, P.1, B.1.617.2, and B.1.1.529 VOCs (recently renamed by WHO as Alpha, Beta, Gamma, Delta and Omicron, respectively) first identified in the United Kingdom, South Africa, Brazil, India, and Botswana, carry different mutations among which D614G change became dominant early in the pandemic [18]. VOCs may increase virus transmissibility, disease severity, reduce neutralization by antibodies generated during previous infection or vaccination, and reduce the effectiveness of treatments or vaccines, or diagnostic detection failures [15,19,20].

Moreover, the large individual variability and the low availability of specific or focused treatments make the use of alternative molecular markers and new therapeutic approaches necessary.

MicroRNAs (miRNAs) are small non-coding RNAs expressed in different tissues and cell types that can interact with target mRNAs, through base-pairing, to inhibit their translation [21]. MiRNAs are powerful regulators of several cellular activities including cell growth, differentiation, development, and apoptosis. An altered miRNA expression is associated with many human diseases [22]. The role of miRNAs in viral infections, including the initiation and progression of infectious diseases, has been reported [23]. It has been demonstrated that virus genomes encode miRNAs (Viral- miRNA, V-miRNA) and, to date, more than 500 V-miRNAs have been reported [24]. V-miRNAs target both viral and cellular transcripts and are involved in cellular reprogramming to regulate the latent-lytic switch, support viral replication by promoting cell survival, proliferation, and/or differentiation, and modulate host immune response [25]. In this way, V-miRNAs and proteins work synergistically, exploiting conserved gene regulatory mechanisms within the host cell to promote a cellular environment favorable to the completion of the viral life cycle [25].

On the other hand, host cellular miRNAs can modulate the expression of viral genes and regulate the tissue tropism of viruses in vivo, playing an important role in host–virus interaction [26]. Cellular miRNAs could interfere or inhibit the viral life cycle representing a defense mechanism for the host [26]. There is some evidence in the literature proving the role of host miRNAs in restricting or promoting the replication of viruses such as Hepatitis B and C, human immunodeficiency virus (HIV), papillomavirus, as well as RNA viruses causing respiratory pathologies such as influenza virus H1N1 and Rhinovirus [27,28,29,30,31]. In the case of HIV infection, host miRNAs are coopted by the virus to fine-tune its replication, keeping it low to escape the immune system and establish a persistent infection [32].

This paper aimed to verify if the SARS-CoV-2 genome is able to produce V-miRNAs showing sequence homology with human miRNAs (hsa-miRNA) and to demonstrate that hsa-miRNAs could regulate the viral genome influencing the cellular response to viral infection. Even if our data are based on a bioinformatics approach, we believe that this study represents a starting point to understand the pathogenic mechanisms of the virus, identify molecular markers of prognosis, and develop new therapeutic strategies.

## 2. Results

### 2.1. Identification of SARS-CoV-2-Derived miRNA Precursors

**Figure 1:**
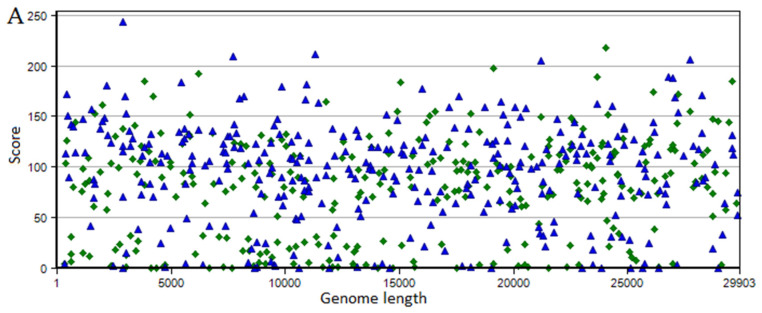

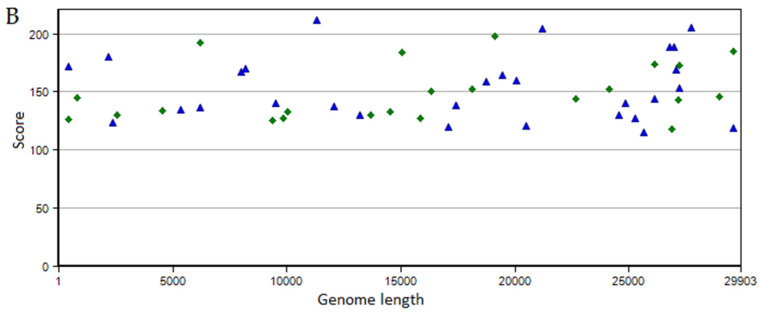
Scatter plot of V-Mir analysis of the SARS-CoV-2 genome: (**A**) V-Mir predictions of all possible V-pre-miRNA hairpins. The hairpin is plotted according to the positions of the viral genome with default parameters with a window size of 500 nt and a step size of 10 nt. (**B**) Customized view scatter plot of SARS CoV-2-predicted V-pre-miRNA hairpins under filtering: 115 minimum hairpin score, windows count value 25, minimum hairpin size 60 nucleotides, maximum hairpin size 120 nucleotides. Hairpins with direct or reverse orientation are shown as blue triangles and green rhombuses.

Searches for SARS-CoV-2-derived miRNA precursors resulted in 663 hairpins and 451 sub-hairpins in the VMir Viewer using default parameters (Figure 1A). The results were further filtered (see Section 4), and 52 probable hairpin candidates were returned with “forward” and “reverse” directions (Figure 1B).

### 2.2. Identification of SARS-CoV-2 Mature miRNAs

By method I, eleven mature V-miRNAs, showing high similarity to hsa-miRNAs previously deposited in miRbase, were obtained from 10 V-pre-miRNAs by the SSEARCH option. Two miRNAs (miR-2114-5p and miR-5680) exhibit 100% identity of the seed region with the viral hairpin pairing sequence, six miRNAs (miR-411-5p, miR-548au-3p, miR-548q, miR-5683, miR-6853-3p, and miR-6867-5p) showed a single mismatch, and three miRNAs (miR-411-5p, miR-548b-5p, and miR-1267) showed two mismatches within the seed region n (Table 1).

Nine mature V-miRNAs were obtained from seven V-pre-miRNAs by the BLASTN option of miRBase. Among these, five miRNAs (miR-190b-5p, miR-744-3p, miR-4699-3p, miR-6730-5p, and miR-6838-5p) showed one mismatch, while the other four miRNAs (miR-181b-3p, miR-545-3p, miR-4420, and miR-5011-3p) showed two mismatches in the pairing region between the miRNA seed sequence and the viral hairpins (Table 1).

By method II, 104 mature V-miRNAs of ≃22 nt in length were identified by the MatureBayes program. For each viral miRNA, sequences of both 5′ and 3′ arms were predicted. Using the SSEARCH option of miRBase, 14 mature viral miRNAs showing high similarity to human miRNAs listed in miRbase were obtained from 10 viral hairpins. Three miRNAs (miR-519c-3p, miR-5680, and miR-6074) showed 100% identity of the seed region with the predicted V-miRNA. Eight miRNAs (miR-147b-5p, miR-365-5p, miR-511-3p, miR-548au-3p, miR-548q, miR-548v, miR-6715b-5p, and miR-6853-3p) displayed one mismatch and three miRNAs (miR-105-3p, miR-147b-5p, and miR-4471) showed two mismatches within the seed region (Table 2). Using the BLASTN option, three mature viral miRNAs derived from four hairpins showed similarity to three human miRNAs. One miRNA (miR-744-3p) exhibited one mismatch and two miRNAs (miR-153-5p and miR-411-5p) showed two mismatches in the pairing region between the miRNA seed sequence and the predicted V-miRNA (Table 2).

**Figure 2:**
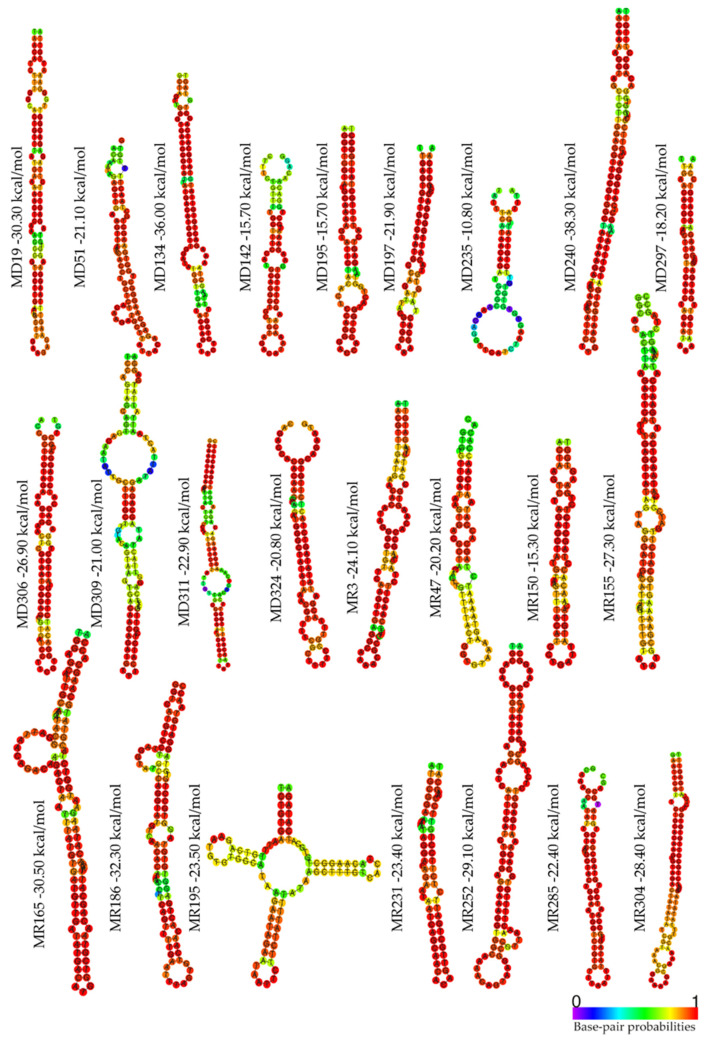
Predicted centroid secondary structures of potential SARS-CoV-2 V-pre-miRNAs.

Finally, we predicted the centroid secondary structure and performed the MFE calculation for each V-pre-miRNA. MFE values calculated for each viral hairpin ranged from −10.80 kcal/mol to −38.30 kcal/mol (Figure 2).

### 2.3. Prediction of Biological Pathways Regulated by SARS-CoV-2 miRNAs

**Figure 3:**
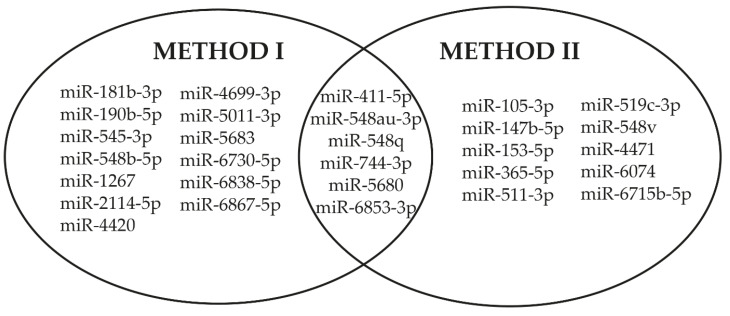
Comparison of the results obtained by two different bioinformatics methods.

The analysis performed on the SARS-CoV-2 genome with the two methods revealed a total of 29 V-miRNAs potentially deriving from viral pre-miRNA sequences. Subsequently, the comparison of the results obtained by the two bioinformatics analyses allowed us to highlight six V-miRNAs that are common to both methods: miR-411-5p, miR-548au-3p, miR-548q, miR-744-3p, miR-5680, and miR-6853-3p (Figure 3).

**Figure 4:**
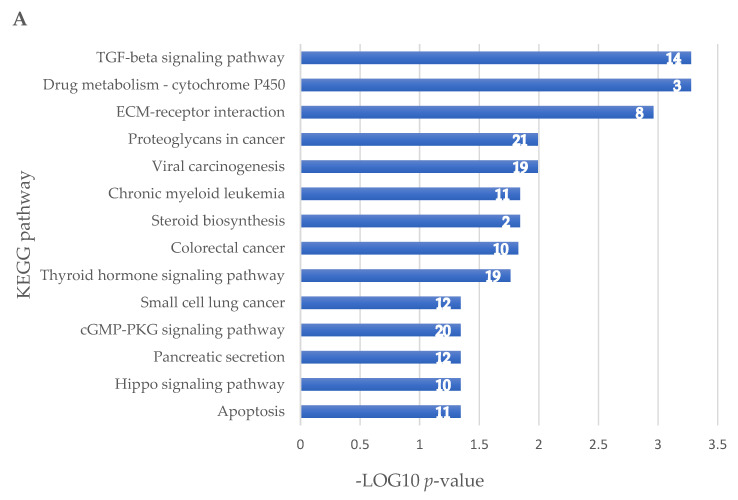

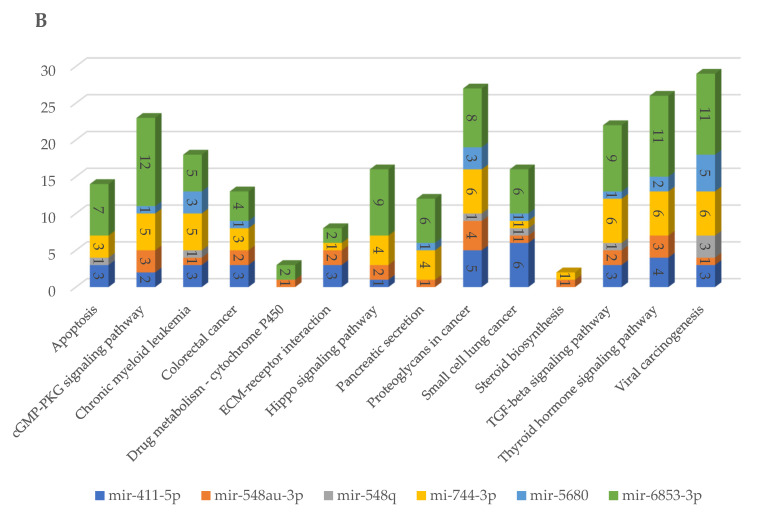
KEGG pathway enrichment analysis. (**A**) The signaling pathways and the number of mRNA targets are shown. The *x*-axis represents the −log10 (*p*-value). (**B**) The figure shows the number of targets for single miRNA in the different pathways.

The six selected miRNAs are able to regulate some cellular pathways such as apoptosis, carcinogenic, inflammatory, and fibrotic processes, as well as small-cell lung cancer (Figure 4A), by targeting different genes (Figure 4B). The results of all target genes according to each miRNA are given in Table S1.

### 2.4. Identification of Human miRNAs Targeting SARS-CoV-2-Specific Genome Sequences

We identified 54 hsa-miRNAs capable of targeting the SARS-CoV-2 genome. Among them, we selected 47 miRNAs that were predicted to potentially bind the Wuhan SARS-CoV-2 genome with a miRDB target score greater than 85 (Table S2). Specifically, we found 12 miRNAs targeting the gene encoding for “S”, 3 miRNAs for “N”, 9 miRNAs for NSP13, 6 miRNAs for NSP14, 3 miRNAs for NSP15, 5 miRNAs for ORF6, 5 miRNAs for ORF7a, 2 miRNAs for ORF7b, and 2 miRNAs for the 5′UTR sequence.

**Figure 5:**
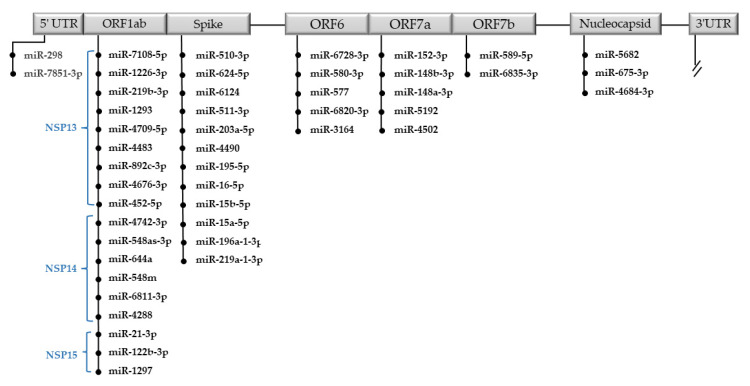
Predicted hsa-miRNAs targeting the single sequences of the SARS-CoV-2 genome. MiRNAs are ordered by the pairing score.

The 47 miRNAs and their target regions are reported in Figure 5. The MFE in the best seed match calculated for predicted miRNAs by miR-DB ranges from −11.7 kcal/mol to −29.5 kcal/mol (Table S2). Free energy values <−11 correlate with a high probability of hsa-miRNAs of targeting the viral genome.

**Figure 6:**
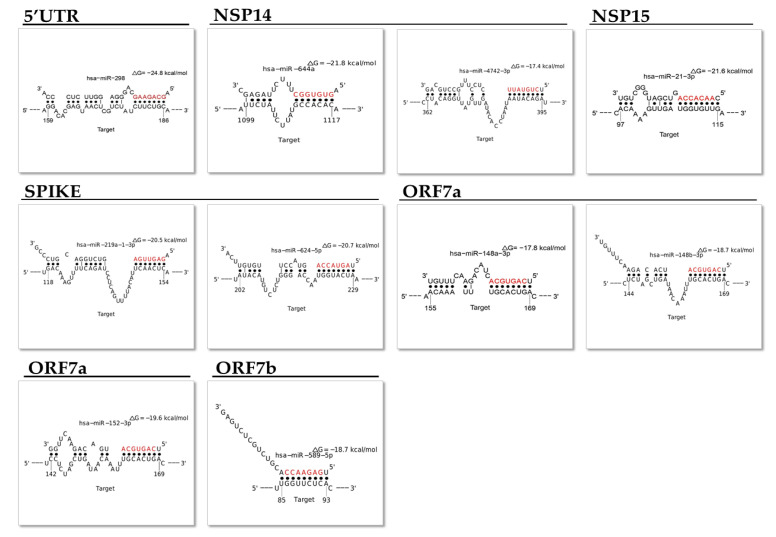
MFE and seed match between hsa-miRNAs and the SARS-CoV-2 genome with the best logit probability score.

Most of the predicted hsa-miRNAs are expressed in different tissues and some of them have been found associated with human diseases. We selected 10 miRNAs expressed in tissues related to COVID-19 and whose altered regulation could be related to the pathogenesis of the disease (Table 3). Figure 6 shows the seed match and MFE between hsa-miRNA and the viral genome with the best logit probability score (Figure 6).

### 2.5. Prediction of Biological Pathways Regulated by miRNAs Able to Target Viral Genome

**Figure 7:**
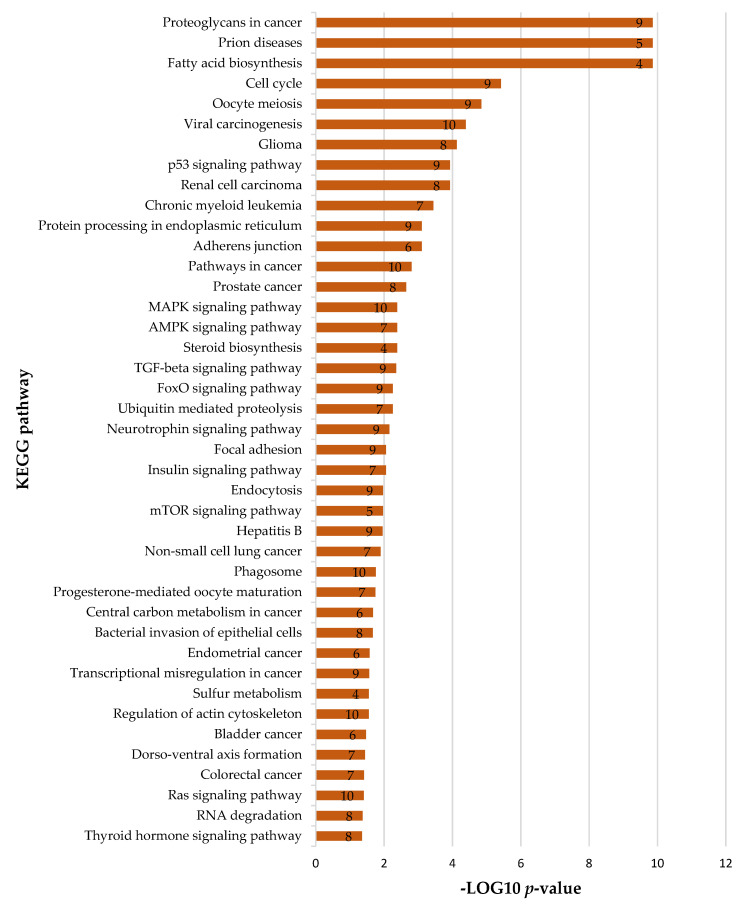
KEGG pathway enrichment analysis for miRNAs expressed in tissues related to COVID-19. The retrieved pathways and the number of mRNA targets are shown. The *x*-axis represents the −log10 (*p*-value).

We identified 42 significant biological pathways regulated by 10 miRNAs that are able to target the SARS-CoV-2 genome and that are expressed in tissues related to COVID-19. These miRNAs regulate some important cellular pathways such as cell cycle, apoptosis, viral carcinogenesis, and proteins that transmit a signal from a receptor to the DNA in the nucleus of the cell such as AMPK, MPK, TGF-beta, FoxO, and p53 (Figure 7).

### 2.6. Analysis of SARS-CoV-2 Variants of Concern (VOCs)

**Figure 8:**
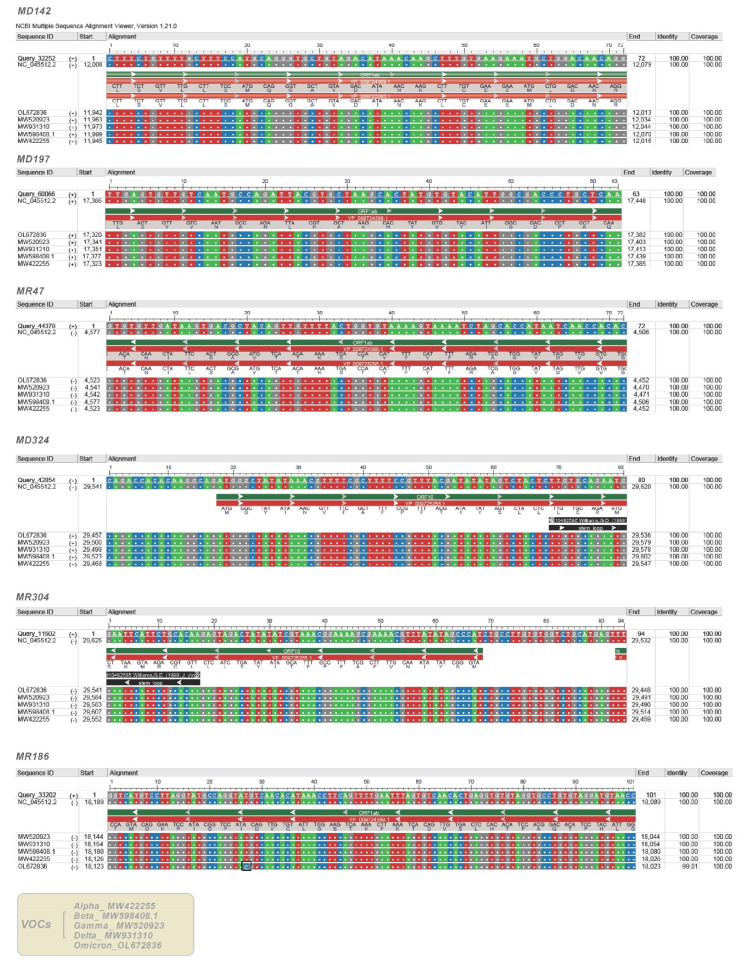
Alignments of predicted viral hairpins to Wuhan SARS-CoV-2 genome and variants of concern (VOCs).

**Figure 9:**
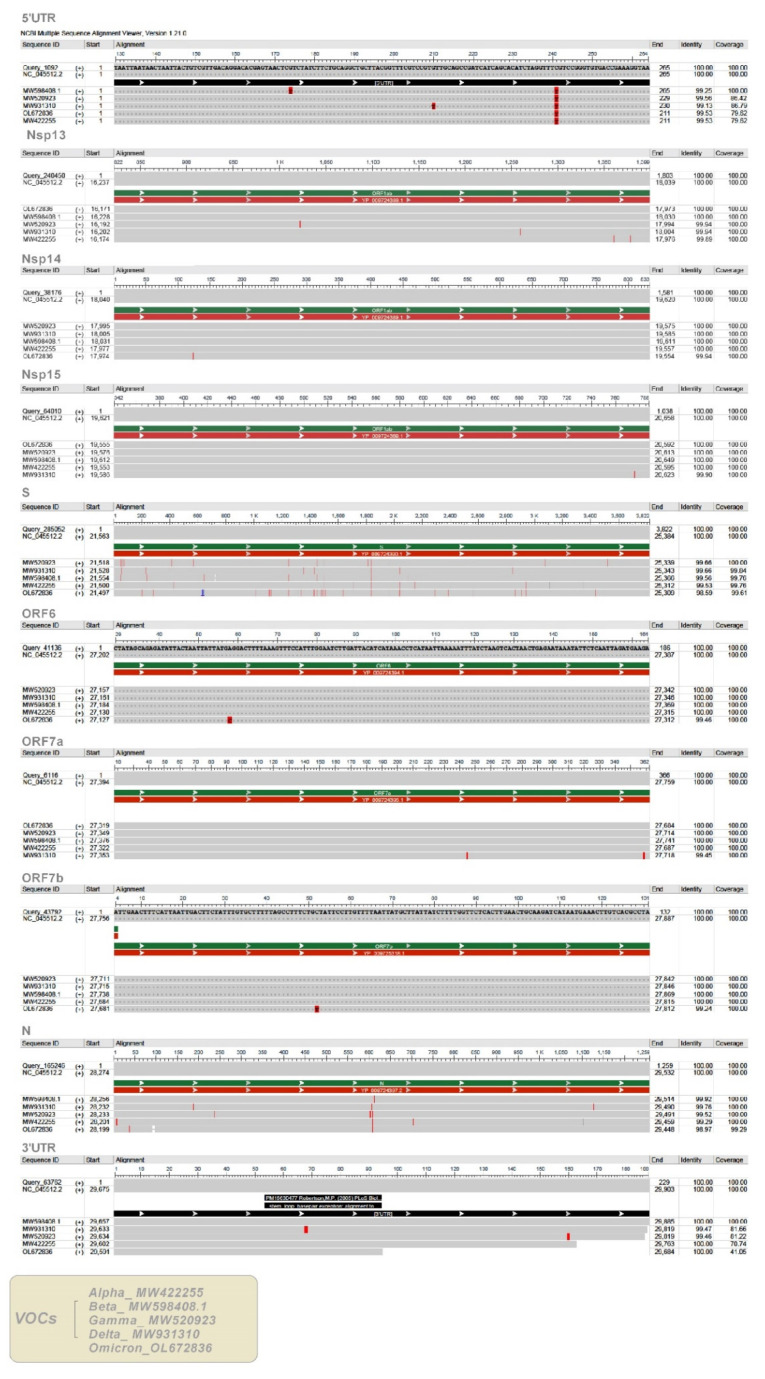
Alignments of Wuhan SARS-CoV-2 genome regions to variants of concern (VOCs).

Alignment analysis of precursors of the six SARS-CoV-2-derived miRNAs, common to both methods, showed a very high nucleotide sequence homology (100% identity and 100% coverage) with four SARS-CoV-2 VOCs (Alpha, Beta, Gamma, and Delta) (Figure 8). A lower sequence homology (99%), but not significant, was observed, instead, about the MR186 precursor with the VOC Omicron. Alignments of the five VOCs with specific regions of the Wuhan SARS-CoV-2 genome are shown in Figure 9. Computational analysis has identified miR-7851-3p capable of binding with a high affinity to the 5′UTR of the Wuhan SARS-CoV-2 genome. Moreover, it has been found that miR-122 and miR-21-3p target S of the beta and gamma variants, miR-182-3p and miR-597-3p S of the delta variant, miR-497-5p and miR-6838-5p S of the gamma variant with the highest miRDB scores, and finally, miR-615-5p target N of alpha with the highest affinity (Table 4). All data are shown in Supplementary Table S3.

## 3. Discussion

In this paper, we aimed to investigate miRNA-mediated regulation in the host cells infected by SARS-CoV-2. We focused our attention on V-miRNAs able to regulate cellular mRNAs and on hsa-miRNAs able to bind the RNA virus genome. By in silico analysis, we found six miRNAs that could be produced from the genome of SARS-CoV-2 and that showed very high homology with human miRNAs. Moreover, we describe hsa-miRNAs able to bind specific regions of the SARS-CoV-2 genome and that could be involved in the cellular response to viral infection. The data could help to understand the pathogenesis of COVID-19 and suggest innovative therapies based on the use of these small non-coding RNAs.

It has long been proven that DNA viruses encode miRNAs, and more than 500 viral miRNAs have been identified, in herpesviruses, polyomaviruses, ascoviruses, and adenoviruses [24]. Even though the majority of viral miRNAs found are encoded by DNA viruses, to date, miRNAs encoded by viruses with RNA genomes have been recorded in an increasing number of studies [33,34,35,36]. The presence of V-miRNAs sharing sequences with hsa-miRNAs can be justified considering the possibility of horizontal transfer of genetic material from viruses to cells and from cells to viruses [37]. Another possibility could be the hypothesis of, still much debated today, giant viruses that could be included in the domains of life [38,39].

By an in silico study, we identified six miRNAs, described in our species, which could be produced by the SARS-CoV-2 viral genome. Previous papers, using bioinformatic analysis, reported that the SARS-CoV-2 genome is able to produce miRNAs [40,41]. In order to identify the most likely V-miRNAs, we performed a stringent approach using two different prediction methods to verify the homology between the predicted V-pre-miRNAs and hsa-miRNAs. Later, we selected the V-miRNAs, miR-411-5p, miR-548au-3p, miR-548q, miR-744-3p, miR-5680, and miR-6853-3p, common to both methods (Figure 3). The selected miRNAs were identified for the first time, except miR-411 and miR-744-3p, which had already been predicted [40,41]. MiR-411 and miR-744-3p overexpression has been reported in cells infected by Hantaan virus (HTNV) and Dengue virus (DENV), respectively [42,43]. Therefore, V-miRNAs could be encoded by SARS-CoV-2 in human cells when infected, which results in miRNAs overexpression.

Moreover, predicted V-miRNAs presented a MFE score ranging from −10.80 kcal/mol to −38.30 kcal/mol (Figure 2) and complete sequence similarity of the seed region with hsa-miRNAs or a maximum of two mismatches (Table 1 and Table 2).

In general, viral miRNAs and cellular miRNAs do not bear seed homology. However, presumably, due to the presence of highly evolved gene-regulatory networks, some viral miRNAs have seed homology with cellular miRNAs [26].

V-miRNAs, sharing the seed sequence with human miRNAs, could act on human mRNA targets; accordingly, V-miRNA expression, in virus-infected cells, could affect cellular pathways concurring to clinical manifestations of COVID-19. The V-miRNAs involved in the different pathways, as well as the mRNAs, are shown in Figure 4. Among the most significant cellular pathways regulated by mRNA targets, we found apoptosis, cGMP-PKG, TGF-beta, and TNF signaling, as well as ECM–receptor interaction and viral carcinogenesis. It has been proposed that V-miRNAs, targeting cellular genes involved in cell proliferation and survival, stress responses and apoptosis, could enhance cell survival, evade the immune response of host cells, and promote viral replication and infection [25].

Recent studies have demonstrated that miR-411 is involved in cancer metastases, and its overexpression in non-small-cell lung cancer cells could inhibit cell proliferation and invasion and promote apoptosis [44,45].

**Figure 10:**
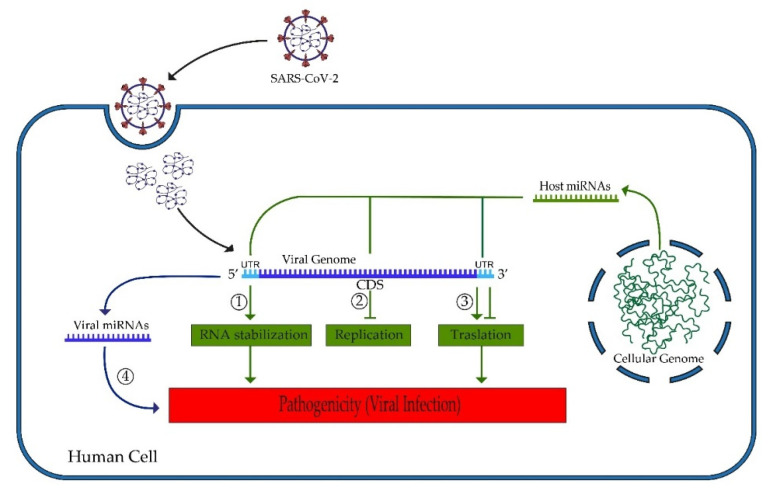
MicroRNAs and SARS-CoV-2 pathogenic mechanisms. Host miRNAs control SARS-CoV-2 RNA genome via targeting of the 5′UTR, stabilizing the RNA (1); the CDS regions, inhibiting viral replication (2); the 3′UTR, inhibiting or inducing translation (3). SARS-CoV-2 miRNAs can alter host response and viral proliferation by regulating different cellular pathways (4).

The second analysis presented in this study concerns the possibility that hsa-miRNAs are able to bind to specific and important sequences of the SARS-CoV-2 genome. Host miRNAs are able to regulate the pathogenesis of the positive-strand RNA virus genome by binding specific regions [46] (Figure 10). Host miRNAs could negatively regulate virus replication by inhibiting the translation of the viral genome; alternatively, the binding to 5′ or 3′UTR could lead to greater RNA stability and increased viral replication [46].

Cellular miRNAs can facilitate the virus replication by targeting the viral 5′ non-coding region or by controlling (repressing) the translation of viral mRNA into protein to allow the virus to escape the host–immune system, which occurs in herpesvirus infection [25,26,47].

Various papers, using in silico studies, have demonstrated that hsa-miRNA can bind the SARS-CoV-2 genome [48]. To verify this possibility and perform more stringent research, we focused our attention on 3′UTR and 5′UTR, canonical binding sites for hsa-miRNAs, on the “S gene” codifying the protein spike and the “gene N” coding the nucleocapsid protein. Moreover, we considered the “gene ORF6”, “CDS NSP13”, “CDS NSP14”, and “CDS NSP15”, able to powerfully suppress both the production of the primary interferon and the signaling cascade of the interferon, and “ORF7a” and “ ORF7b”, accessory genes with a role not yet entirely clear [49]. The 47 identified miRNAs and the possible target genes are reported in Figure 5. Some of them are expressed in tissues (organs) mainly involved in viral infection such as the lung, heart, and liver, and their altered regulation is related to lung cancer, asthma, and autoimmune disorders of the central nervous system (Table 3 and Figure 7). Considering that subjects affected by these diseases have a worse prognosis when they contract COVID-19, it could be hypothesized that the altered expression of miRNAs could cause an inadequate cellular response to virus infection. We found that hsa-miR-219 could target the spike protein; its down-regulation could explain the high severity of COVID-19 in non-small cell lung cancer patients [50]. Likewise, hsa-miR-298 can bind the 5′UTR region, and its down-regulation in Alzheimer disease has been demonstrated. According to previously published papers, the binding of miRNAs to the viral genome may reduce the levels of free miRNAs in the cell, and it has been recently reported that COVID patients may develop an Alzheimer’s-like disease [51]. Moreover, the ability of hsa-miR-3941 to target the SARS-CoV-2 3′-UTR was recently validated in vitro by gene reporter assays [52]. The antiviral effects of miR-122 and miR-148-3p against HCV virus infections have been shown [53,54]. miR-122 was proposed as a therapeutic target against chronic HCV infections and a potential biomarker of hepatic damage by the virus [55]. The modulation of susceptibility to HIV-1 infection, by the targeting of purine-rich element binding protein alpha (Pur-α) mRNA, was also recently demonstrated for miR-15a, miR-15b, and miR-16 [56].

Infections are among the major selective forces acting on humans, and host–pathogen interactions contribute to shaping the genetic diversity of both organisms. Protein regions at the host–pathogen interface are expected to be targeted by the strongest selective pressure [57]. SARS-CoV-2 evolution has been characterized by the emergence of sets of mutations, in the context of “variants of concern”, that impact virus characteristics, including transmissibility and antigenicity, probably in response to the changing immune profile of the human population [16].

Validation analyses considering the five SARS-CoV-2 VOCs indicate that none of the variants present mutations such as to affect viral hairpins derivable from the SARS-CoV-2 genome. Therefore, the five VOCs explored could encode all V-miRNAs predicted. Moreover, most of the variants retain the binding sites for hsa-miRNAs predicted to target Wuhan SARS-CoV-2 RNA. Interestingly, only miR-7851-3p binds with high affinity to the 5′UTR of the Wuhan SARS-CoV-2 genome. This could be due to the alignment to incomplete sequences of VOCs. MiR-7851-3p has been reported to recognize structures that are highly conserved among other viruses in the Coronaviridae family and are important for viral function [58]. Thus, another explanation could be that viruses under selective pressure could avoid functional binding sites for miRNAs, which are abundantly expressed in their normal target cells, and viral genomes are likely optimized for replication in that environment. This hypothesis suggests that the fortuitous repression of viral mRNAs by cellular miRNAs in nontarget tissues could represent a significant hurdle to successful infection and thereby might serve as a key determinant of viral tissue tropism [59]. Computational analysis has identified miRNAs that could most likely target only one or more VOCs on the RNA sequence encoding the spike protein of the virus and that could have epidemiological importance in SARS-CoV-2 infections. For example, miR-182-3p targets the Delta variant with a score of 90, while it binds Alpha, Beta, Gamma, Omicron, and Wuhan SARS-CoV-2 RNA with a score of 57. The lack of conservation of miRNA target sites in viral sequences linked to the higher rate of mutations and faster evolution in viruses could mean an evolutionary advantage for rapid adaptation to the host and environmental conditions [26].

## 4. Materials and Methods

### 4.1. Prediction of SARS-CoV-2-Derived miRNA Precursors (pre-miRNAs) Specific Genome Sequences

**Figure 11:**
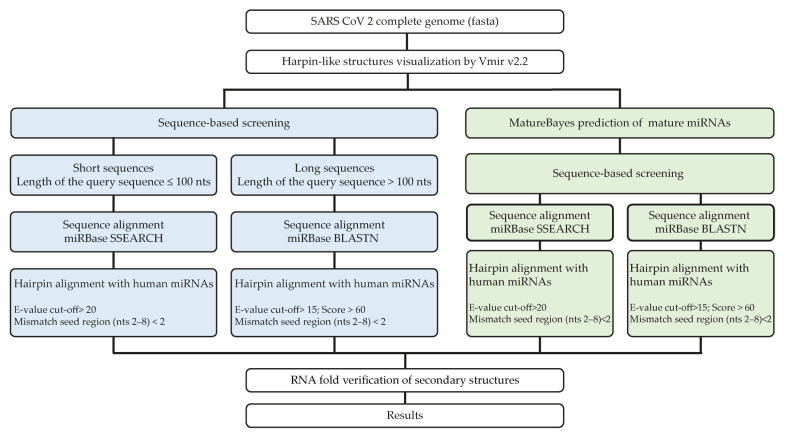
miRNA prediction from the SARS-CoV 2 genome. Workflow of the two methods used to predict the sequences of V-miRNAs showing homology with hsa-miRNAs. Blue squares show method 1 of analysis, while green squares show method 2 of analysis.

The reference genome sequence of SARS-CoV-2 novel coronavirus (NC_045512.2) isolated in Wuhan [60] was retrieved from the genome database of the National Center for Biotechnology Information (NCBI) and used for our analysis. First, a computational prediction step was conducted to identify virus-derived miRNA precursors (pre-VmiRNAs), hairpin-structured. The SARS-CoV-2 genome was scanned using the VMir software package, an ab initio prediction program that was specifically designed to identify pre-miRNA structures in the viral genome [61]. The VMir Analyzer (v2.2) prediction tool was set using the default parameters: sequence conformation “linear”, sequence orientation “both”, folding window size “500” and step size “10”, minimum harpin size “50”, maximum harpin size “any”, and minimum harpin score “any.” Results were then observed using the VMir Viewer (v1.5) visualization tool with customized settings: 115 minimum hairpin score, windows count value 25, minimum hairpin size 60 nucleotides, maximum hairpin size 120 nucleotides. The potential hairpin structures were extracted for subsequent analyses. Inside these structures, we looked for sequences having high similarity with hsa-miRNAs, by using two different methods (Figure 11).

### 4.2. Methods for Selection of V-miRNAs

In the first method, the viral hairpins predicted by VMir were divided into short sequences (nucleotide number equal to or less than 100 nts) and long sequences (nucleotide number greater than 100 nts) and given as an input to miRBase (www.mirbase.org/search.shtml) (accessed on 20 November 2021) for sequence similarity analysis. The short sequences were analyzed by the SSEARCH algorithm and the long sequences by the BLASTN algorithm (Figure 11, blue squares).

In the second method, from the viral hairpins, mature V-miRNAs were predicted using the web tool MatureBayes (http://mirna.imbb.forth.gr/MatureBayes.html (accessed on 3 December 2021)). MatureBayes is a computational algorithm that uses a Naive Bayesian classifier to predict the start position of the mature miRNA on human and mouse miRNA precursors [62]. MatureBayes returns the probable mature sequences including both 3′ and 5′ mature miRNAs. The predicted V-miRNAs were analyzed by both SSEARCH and BLASTN, to find V-miRNAs sharing sequences with hsa-miRNAs (Figure 11, green squares).

For both methods, we selected the alignments in which the viral sequences show a maximum of two mismatches with the hsa-miRNA seed region. For the alignments carried out with BLASTN, the length of the pairing between the two sequences was also taken into consideration, and 18 nucleotides were set as the minimum value to identify a BLAST match. Moreover, a cut-off score of 60 and an e-value threshold of 15 were applied. For the analyses carried out with the SSEARCH algorithm, an e-value threshold of 20 was used.

Finally, the viral hairpins identified through the different methods were further analyzed for centroid secondary structure prediction by the RNA fold web server (http://rna.tbi.univie.ac.at/cgi-bin/RNAWebSuite/RNAfold.cgi (accessed on 3 December 2021)) and the minimum free energy (MFE) calculation.

### 4.3. Prediction of Molecular Pathway Regulated by V-miRNAs

The KEGG (Kyoto Encyclopedia of Genes and Genomes) molecular pathways regulated by mature SARS-CoV-2 miRNAs were explored by using the DIANA-miRPath v.3 webserver (http://diana.imis.athena-innovation.gr/DianaTools/index.php (accessed on 3 December 2021)) by setting the search for experimentally validated miRNA gene targets.

MiR-6853-3p does not have any annotated targets in the TARBASE database, because it is a recently discovered miRNA. We used microT-CDS, an algorithm for the identification of predicted targets, and selected those having a microT threshold of 0.85. The FDR method was implemented to select the biological pathways with a threshold of significance defined by *p* < 0.05.

### 4.4. Prediction of Human miRNAs Targeting the SARS-CoV-2 Genome

Genomic sequences of SARS-CoV-2 used to identify target sites of human miRNA were obtained from NCBI (https://www.ncbi.nlm.nih.gov/nuccore/NC_045512 (accessed on 3 December 2021)). We selected specific genome sequences according to their role in the virus life cycle and COVID-19 pathogenesis. Specifically, we considered the genes encoding the spike and nucleocapsid proteins, some ORFs (1ab “NSP13, NSP14, and NSP15”, 6, 7a, 7b), and the 5′ and 3′UTR [49].

The miRDB web server (http://mirdb.org (accessed on 3 December 2021)) was used to predict the target sites of hsa-miRNAs within viral sequences. A cut-off ≥85 was set in order to consider only the most significant results. The free energy of the best match in seed regions was calculated by StarMir (https://sfold.wadsworth.org/cgi-bin/starmirtest2.pl (accessed on 3 December 2021)).

### 4.5. Prediction of Tissue-Specific Expression of miRNAs and Their Involvement in Human Disease

Gene cards database version 5.3 (https://www.genecards.org/ (accessed on 3 December 2021)) was used to explore human tissues in which hsa-miRNAs targeting the viral genome are expressed and to retrieve information on pathologies with which the hsa-miRNAs could be associated.

### 4.6. Prediction of Molecular Pathways Regulated by hsa-miRNAs

The KEGG (Kyoto Encyclopedia of Genes and Genomes) molecular pathways regulated by human miRNAs targeting the viral genome were explored by using the DIANA-miRPath v.3 webserver (http://diana.imis.athena-innovation.gr/DianaTools/index.php (accessed on 3 December 2021)), setting the search for experimentally validated miRNA target genes.

MiR-152-3p, miR-219a-1-3p, and miR-644a do not have any annotated targets in the TARBASE database, so we used microT-CDS, an algorithm for the identification of predicted targets, and selected those having a microT threshold of 0.85.

The FDR method was implemented to select the biological pathways with a threshold of significance defined by *p* < 0.05.

### 4.7. Analysis of SARS-CoV-2 Variants of Concern (VOCs)

We tested the sequences related to viral hairpins by alignment to 5 SARS-CoV-2 variants of concern (VOCs), identified in the United Kingdom (Alpha) (B.1.1.7), South Africa (Beta) (B.1.351), Brazil (Gamma) (P.1), India (Delta) (B.1.617.2), and Botswana (Omicron) (B.1.1.529), in which the spike protein mutation D614G became dominant during the COVID-19 pandemic. We retrieved the genome sequences of the 5 SARS-CoV-2 VOCs (MW422255, MW598408.1, MW520923, MW931310, and OL672836) from NCBI SARS-CoV-2 Resources https://www.ncbi.nlm.nih.gov/sars-cov-2/ (accessed on 3 December 2021). We used the multiple alignment search tool BLAST (National Center for Biotechnology Information (NCBI)) and visualized the aligned sequences using the web application Multiple Sequence Alignment Viewer 1.21.0. Then, we performed multiple alignments between the specific regions of the Wuhan virus genome, targets of the predicted human miRNAs, and VOCs to validate human miRNAs regulation on the viral genome. The miRDB web server was used to predict the target sites of hsa-miRNAs within VOCs.

## 5. Conclusions

COVID-19 represents a public health emergency, whose pathophysiology is not fully understood. Virus and host microRNAs may play a crucial role in host cells after infection by SARS-CoV-2, contributing to severe forms of the disease. There appears to be no difference in the SARS-CoV-2 VOCs analyzed from which the predicted viral miRNAs could derive. Furthermore, some of the human miRNAs that are capable of binding more strongly to some variants remain to be considered and validated.

These data could help to understand the pathogenesis of COVID-19 and suggest innovative therapies based on the use of these small non-coding RNAs.

## Figures and Tables

**Table 1:** Human miRNAs (hsa-miRNAs) with sequence homology to SARS-CoV2 V-pre-miRNAs. The number of allowed mismatches between the V-pre-miRNAs and the seed region (SR) is shown.

SSEARCH			BLASTN		
Viral-Hairpins	Hsa-miRNAs	SR Mismatch	Viral-Harpins	Hsa-miRNAs	SR Mismatch
MD306	miR-2114-5p	0	MD134	miR-190b-5p	1
MD142	miR-5680	0	MR186	miR-744-3p	1
MR304	miR-411-5p	1	MD311	miR-4699-3p	1
MR47	miR-548au-3p	1	MD19	miR-6730-5p	1
	miR-548q	1	MD309	miR-6838-5p	1
MR231	miR-5683	1	MD309	miR-181b-3p	2
MD197	miR-6853-3p	1	MR252	miR-545-3p	2
MD297	miR-6867-5p	1	MR186	miR-4420	2
MR231	miR-5683	1	MD240	miR-5011-3p	2
MD324	miR-411-5p	2			
MD195 MD324	miR-548b-5p	2			
MD51	miR-1267	2			

**Table 2:** Human miRNAs with high similarity to SARS-CoV2 mature V-miRNA sequences. Seed region (SR) mismatch indicates the mismatch value of the human miRNA seed region with the possible V-miRNA.

SSEARCH			BLASTN		
Viral-Hairpins	Hsa-miRNAs	SR Mismatch	Viral-Harpins	Hsa-miRNAs	SR Mismatch
MR155	miR-519c-3p	0	MR186	miR-744-3p	1
MD142	miR-5680	0	MR165	miR-153-5p	2
MR150	miR-6074	0	MD324	miR-411-5p	2
MR252	miR-147b-5p	1	MR304		2
MR155	miR-365-5p	1			
MR195	miR-511-3p	1			
MR47	miR-548au-3p	1			
	miR-548q	1			
	miR-548v	1			
MR285	miR-6715b-5p	1			
MD197	miR-6853-3p	1			
MD235	miR-105-3p	2			
MD142	miR-147b-5p	2			
MR3	miR-4471	2			

**Table 3:** MiRNAs expressed in tissues related to COVID-19 and whose altered regulation could be related to the pathogenesis of the disease. Acute Lymphocytic Leukemia: ALL; Central Nervous System tumors: CNS tumors; Cholangiocarcinoma: CCA; Esophageal Cancer: ESCR; Hepatocellular Carcinoma: HCC; Lung Cancer: LC.

Hsa-miRNA	Tissue	Disease	Target Sequence	Score
miR-298	Adrenal glans, Arteries, Heart, Lung	Alzheimer’s Disease	5′UTR	91
miR-644a	Arteries, Brain, Cortex Cerebellum, Heart, Lung		NSP14	92
miR-4742-3p	Adrenal glands, Brain, Cerebellum Cortex, Heart, Liver, Lung, Kidney		NSP14	94
miR-21-3p	Arteries, Blood, Lung, Thyroid	Cholesteatoma, Glioma, Larynx cancer, Oral Squamous Cell Carcinoma, Tongue Squamous Cell Carcinoma	NSP15	94
miR-219a-1-3p	Adrenal glands, Brain, Cortex Cerebellum, Heart, Kidney, Lung	CNS tumors, HCC, Infratentorial cancer, LC, Sacral cordoma	SPIKE	85
miR-624-5p	Brain, Cerebellum, Heart, Lung		SPIKE	90
miR-148a-3p	Blood, Liver, Lymphnodes, Intestines	Asthma, CCA, ESCR	ORF7a	94
miR-148b-3p	Adrenal glands, Brain, Heart, Lung	Asthma, Oral Squamous Cell Carcinoma	ORF7a	94
miR-152-3p	Arteries, Colon, Pituitary gland, Thyroid	ALL, Asthma, CCA, HCC	ORF7a	94
miR-589-5p	Adrenal glands, Heart, Liver, Lung, Nervous tissue		ORF7b	94

**Table 4:** hsa-miRNAs targeting Wuhan SARS-CoV-2 genome regions and VOCs. Predicted miRNAs with miRDB scores (values in brackets) higher than 85 are indicated in bold.

**5′UTR**					
Wuhan	Alpha	Beta	Gamma	Delta	Omicron
**miR-7851-3p (91)**	nd	nd	nd	nd	nd
**SPIKE**					
Wuhan	Alpha	Beta	Gamma	Delta	Omicron
miR-122b-3p (71)	miR-122b-3p (80)	**miR-122b-3p (87)**	**miR-122b-3p (87)**	miR-122b-3p (80)	miR-122b-3p (79)
miR-182-3p (57)	miR-182-3p (57)	miR-182-3p (57)	miR-182-3p (57)	**miR-182-3p (90)**	miR-182-3p (57)
miR-21-3p (71)	miR-21-3p (80)	**miR-21-3p (87)**	**miR-21-3p (87)**	miR-21-3p (80)	miR-21-3p (79)
miR-424-5p (84)	miR-424-5p (84)	miR-424-5p (84)	**miR-424-5p (89)**	miR-424-5p (84)	miR-424-5p (84)
miR-497-5p (84)	miR-497-5p (84)	miR-497-5p (84)	**miR-497-5p (89)**	miR-497-5p (84)	miR-497-5p (84)
miR-597-3p (81)	miR-597-3p (81)	miR-597-3p (81)	miR-597-3p (81)	**miR-597-3p (88)**	miR-597-3p (81)
miR-6838-5p (84)	miR-6835-5p (68)	miR-6835-5p (68)	**miR-6838-5p (89)**	miR-6838-3p (51)	miR-6835-5p (68)
**Nucleocapsid**					
Wuhan	Alpha	Beta	Gamma	Delta	Omicron
miR-615-5p (79)	**miR-615-5p (89)**	miR-615-5p (79)	miR-615-5p (79)	miR-615-5p (79)	miR-615-5p (80)
**3′UTR**					
Wuhan	Alpha	Beta	Gamma	Delta	Omicron
miR-3941 (84)	miR-3941 (85)	miR-3941 (85)	miR-3941 (85)	miR-3941 (85)	miR-3941 (80)

## Supplementary Materials

The following are available online at https://www.mdpi.com/article/10.3390/ijms222413192/s1.
