# Supplementary material for: MicroRNA-Mediated Regulation of the Virus Cycle and Pathogenesis in the SARS-CoV-2 Disease
Source: Int J Mol Sci. 2021 Dec 7;22(24):13192. doi: 10.3390/ijms222413192 (PMC8715670; doi:10.3390/ijms222413192)
Supplement: Supplementary file 1 [file ijms-22-13192-s001.zip › ijms-1440854-SI.pdf]

International Journal of *Molecular Sciences*

MDPI

Article

MicroRNA-mediated regulation of the virus cycle and pathogenesis in the SARS-CoV-2 disease

Rosalia Battaglia, Ruben Alonzo, Chiara Pennisi, Angela Caponnetto, Carmen Ferrara, Michele Stella, Cristina Barbagallo, Davide Barbagallo, Marco Ragusa, Michele Purrello and Cinzia Di Pietro *

Supplementary Table S1. Validated target genes, **from** KEGG analysis, of six V-miRNAs: miR-411-5p, miR-548au-3p, miR-548q, miR-744-3p, miR-5680 and miR-6853-3p. Predicted targets and scores by microT-CDS for miR-6853-3p are shown.

miR-411-5p
BIRC3
CCND1
CEBPB
COL4A2
FGF2
FN1
FST
HSP90AB1
NCOA1
PDGFRA
PIK3CB
SMURF2
THBS2

miR-548au-3p
ATP2A3
DHCR24
FMO5
GNAI2
HSPG2
MSH3
MYC
NCOR1
NOTCH2
ROCK2
THBS1
YWHAZ

miR-548q
IGF1R
NFKBIA
SP1
UBE3A

miR-5680
CDK6
GTF2A1
MDM2
RHOA

miR-744-3p
AIFM1
BAG4
CASP3
CDKN1B
CHD4
CREB3
CRK
DAG1
DDX5
INHBB
JUNB
MDM2
MED14
MEF2D
MSH6
MSMO1
NCOA2
NCOA3
PGAM5
PPP1R12A
PPP2CA
PPP3CB
RAF1
SKP1
SMURF2
YAP1

miR-6853-3p	Score	miR-6853-3p	Score
ADH1B	0.928	KCNMA1	0.970
AMOT	0.817	LATS1	0.801
APAF1	0.954	MAP2K4	0.893
ATP1B1	0.991	MAPK14	0.849
ATP1B3	0.872	MECOM	0.865
ATP2A2	0.942	MED1	0.800
ATP2B1	0.989	MEF2A	0.853
ATP2B3	0.816	NCOA2	0.837
ATP6V0D1	0.925	NOG	0.989
BMPR2	0.808	NOS3	0.861
CCR8	0.886	PDGFD	0.840
CDK6	0.802	PIK3CB	0.919
CREB3L1	0.847	PIK3R2	0.881
CSF2RB	0.872	PLCZ1	0.876
CTBP2	0.807	PPP2CA	0.997
CTGF	0.975	PPP3CA	0.891
CTNNA1	0.956	PRKAR2A	0.839
CX3CL1	0.806	RBPJ	0.988
CXCL5	0.824	RPS6KB1	0.997
CYP3A4	0.913	SLC16A10	0.874
DFFA	0.826	SMAD2	0.946
DNM1L	0.908	SMAD7	0.995
EP300	0.946	SMURF2	0.812
FZD3	0.898	TBC1D4	0.915
GUCY1A2	0.831	THBS3	0.879
ID4	0.836	TLR4	0.958
IRAK3	0.943	UBE3A	0.950
IRS1	0.880	USP7	0.985
ITGAV	0.833	WNT9B	0.809

	ITGB1	0.900	YAP1	0.960
	ITGB8	0.820		

Supplementary Table S2. Table showing seed match and MFE between hsa-miRNA and Viral Genome with best LogitProbability score. The miRNAs are ordered by MFE score.

hsa-miRNAs	ΔG°	LogitProb	hsa-miRNAs	ΔG°	LogitProb
miR-7108-5p	-29.5	0.908	miR-15a-5p	-20.1	0.904
miR-15b-5p	-28.9	0.93	miR-152-3p	-19.6	0.759
miR-6124	-26.5	0.888	miR-6728-3p	-19.3	0.902
miR-4684-3p	-25.8	0.894	miR-4709-5p	-19.1	0.848
miR-298	-24.8	0.888	miR-892c-3p	-18.8	0.855
miR-7851-3p	-24.7	0.519	miR-148b-3p	-18.7	0.808
miR-1226-3p	-24	0.888	miR-589-5p	-18.7	0.945
miR-1293	-23.8	0.879	miR-122b-3p	-18.4	0.86
miR-5192	-23.7	0.852	miR-580-3p	-18	0.947
miR-6811-3p	-23.4	0.89	miR-148a-3p	-17.8	0.864
miR-16-5p	-23.4	0.942	miR-548as-3p	-17.6	0.906
miR-4502	-23.1	0.881	miR-548m	-17.5	0.92
miR-203a-5p	-22.7	0.883	miR-4742-3p	-17.4	0.845
miR-219b-3p	-22.5	0.779	miR-675-3p	-16.9	0.851
miR-644a	-21.8	0.939	miR-1297	-16.8	0.84
miR-195-5p	-21.7	0.941	miR-6835-3p	-16.3	0.821
miR-21-3p	-21.6	0.86	miR-3164	-16.1	0.781
miR-4288	-21.5	0.945	miR-452-5p	-15.9	0.89
miR-5682	-21.5	0.801	miR-196a-1-3p	-15.6	0.954
miR-510-3p	-20.9	0.921	miR-4676-3p	-14.9	0.887
miR-624-5p	-20.7	0.875	miR-6820-3p	-13.4	0.783
miR-219a-1-3p	-20.5	0.886	miR-577	-12.2	0.906
miR-4483	-20.3	0.892	miR-511-3p	-11.7	0.809
miR-4490	-20.2	0.941			

Supplementary Table S3. hsa-miRNAs targeting Wuhan SARS-CoV-2 genome regions and VOCs. miRDB scores are shown in brackets.

5'UTR					
Wuhan	Alpha	Beta	Gamma	Delta	Omicron
miR-298 (91)	miR-298 (92)	miR-298 (96)	miR-298 (92)	miR-298 (92)	miR-298 (92)
miR-7851-3p (91)	nd	nd	nd	nd	nd
NSP13					
Wuhan	Alpha	Beta	Gamma	Delta	Omicron
miR-1226-3p (90)	miR-1226-3p (90)	miR-1226-3p (90)	miR-1226-3p (90)	miR-1226-3p (90)	miR-1226-3p (90)
miR-1293 (87)	miR-1293 (87)	miR-1293 (87)	miR-1293 (87)	miR-1293 (87)	miR-1293 (87)
miR-219b-3p (88)	miR-219b-3p (88)	miR-219b-3p (88)	miR-219b-3p (88)	miR-219b-3p (88)	miR-219b-3p (88)
miR-4483 (86)	miR-4483 (86)	miR-4483 (86)	miR-4483 (86)	miR-4483 (86)	miR-4483 (86)
miR-452-5p (86)	miR-452-5p (86)	miR-452-5p (86)	miR-452-5p (86)	miR-452-5p (86)	miR-452-5p (86)
miR-4676-3p (86)	miR-4676-3p (86)	miR-4676-3p (86)	miR-4676-3p (86)	miR-4676-3p (86)	miR-4676-3p (86)
miR-4709-5p (87)	miR-4709-5p (87)	miR-4709-5p (87)	miR-4709-5p (87)	miR-4709-5p (87)	miR-4709-5p (87)
miR-7108-5p (96)	miR-7108-5p (96)	miR-7108-5p (96)	miR-7108-5p (96)	miR-7108-5p (96)	miR-7108-5p (96)
miR-892c-3p (86)	miR-892c-3p (86)	miR-892c-3p (86)	miR-892c-3p (86)	miR-892c-3p (86)	miR-892c-3p (86)
NSP14					
Wuhan	Alpha	Beta	Gamma	Delta	Omicron
miR-4742-3p (94)	miR-4742-3p (94)	miR-4742-3p (94)	miR-4742-3p (94)	miR-4742-3p (94)	miR-4742-3p (94)
miR-548as-3p (94)	miR-548as-3p (94)	miR-548as-3p (94)	miR-548as-3p (94)	miR-548as-3p (94)	miR-548as-3p (94)
miR-644a (92)	miR-644a (92)	miR-644a (92)	miR-644a (92)	miR-644a (92)	miR-644a (92)
miR-548m (88)	miR-548m (88)	miR-548m (88)	miR-548m (88)	miR-548m (88)	miR-548m (88)
miR-6811-3p (86)	miR-6811-3p (86)	miR-6811-3p (86)	miR-6811-3p (86)	miR-6811-3p (86)	miR-6811-3p (86)
miR-4288 (85)	miR-4288 (85)	miR-4288 (85)	miR-4288 (85)	miR-4288 (85)	miR-4288 (85)
NSP15					
Wuhan	Alpha	Beta	Gamma	Delta	Omicron
miR-122b-3p (94)	miR-122b-3p (94)	miR-122b-3p (94)	miR-122b-3p (94)	miR-122b-3p (94)	miR-122b-3p (94)
miR-1297 (85)	miR-1297 (85)	miR-1297 (85)	miR-1297 (85)	miR-1297 (85)	miR-1297 (85)
miR-21-3p (94)	miR-21-3p (94)	miR-21-3p (94)	miR-21-3p (94)	miR-21-3p (94)	miR-21-3p (94)
SPIKE					
Wuhan	Alpha	Beta	Gamma	Delta	Omicron
miR-122b-3p (71)	miR-122b-3p (80)	miR-122b-3p (87)	miR-122b-3p (87)	miR-122b-3p (80)	miR-122b-3p (79)
miR-15a-5p (87)	miR-15a-5p (87)	miR-15a-5p (87)	miR-15a-5p (91)	miR-15a-5p (87)	miR-15a-5p (87)
miR-15b-5p (87)	miR-15b-5p (87)	miR-15b-5p (87)	miR-15b-5p (91)	miR-15b-5p (87)	miR-15b-5p (87)
miR-16-5p (87)	miR-16-5p (87)	miR-16-5p (87)	miR-16-5p (91)	miR-16-5p (87)	miR-16-5p (87)
miR-182-3p (57)	miR-182-3p (57)	miR-182-3p (57)	miR-182-3p (57)	miR-182-3p (90)	miR-182-3p (57)
miR-195-5p (87)	miR-195-5p (87)	miR-195-5p (87)	miR-195-5p (91)	miR-195-5p (87)	miR-195-5p (87)
miR-196a-1-3p (86)	miR-196a-1-3p (86)	miR-196a-1-3p (86)	miR-196a-1-3p (86)	miR-196a-1-3p (86)	miR-196a-1-3p (86)

miR-203a-5p (87)	miR-203a-5p (87)	miR-203a-5p (87)	miR-203a-5p (87)	miR-203a-5p (87)	miR-203a-5p (87)
miR-21-3p (71)	miR-21-3p (80)	miR-21-3p (87)	miR-21-3p (87)	miR-21-3p (80)	miR-21-3p (79)
miR-219a-1-3p (85)	miR-219a-1-3p (85)	miR-219a-1-3p (85)	miR-219a-1-3p (85)	miR-219a-1-3p (85)	miR-219a-1-3p (85)
miR-424-5p (84)	miR-424-5p (84)	miR-424-5p (84)	miR-424-5p (89)	miR-424-5p (84)	miR-424-5p (84)
miR-4490 (87)	miR-4490 (87)	miR-4490 (87)	miR-4490 (87)	miR-4490 (87)	miR-4490 (87)
miR-497-5p (84)	miR-497-5p (84)	miR-497-5p (84)	miR-497-5p (89)	miR-497-5p (84)	miR-497-5p (84)
miR-510-3p (92)	miR-510-3p (92)	miR-510-3p (92)	miR-510-3p (92)	miR-510-3p (92)	miR-510-3p (92)
miR-511-3p (89)	miR-511-3p (89)	miR-511-3p (89)	miR-511-3p (89)	miR-511-3p (89)	miR-511-3p (89)
miR-597-3p (81)	miR-597-3p (81)	miR-597-3p (81)	miR-597-3p (81)	miR-597-3p (88)	miR-597-3p (81)
miR-6124 (89)	miR-6124 (89)	miR-6124 (89)	miR-6124 (89)	miR-6124 (89)	miR-6124 (87)
miR-624-5p (90)	miR-624-5p (90)	miR-624-5p (90)	miR-624-5p (90)	miR-624-5p (91)	miR-624-5p (90)
miR-6838-5p (84)	miR-6835-5p (68)	miR-6835-5p (68)	miR-6838-5p (89)	miR-6838-3p (51)	miR-6835-5p (68)
ORF6a					
Wuhan	Alpha	Beta	Gamma	Delta	Omicron
miR-3164 (86)	miR-3164 (86)	miR-3164 (86)	miR-3164 (86)	miR-3164 (86)	miR-3164 (86)
miR-577 (89)	miR-577 (89)	miR-577 (89)	miR-577 (89)	miR-577 (89)	miR-577 (89)
miR-580-3p (94)	miR-580-3p (94)	miR-580-3p (94)	miR-580-3p (94)	miR-580-3p (94)	miR-580-3p (94)
miR-6728-3p (96)	miR-6728-3p (96)	miR-6728-3p (96)	miR-6728-3p (96)	miR-6728-3p (96)	miR-6728-3p (96)
miR-6820-3p (86)	miR-6820-3p (86)	miR-6820-3p (86)	miR-6820-3p (86)	miR-6820-3p (86)	miR-6820-3p (86)
ORF7a					
Wuhan	Alpha	Beta	Gamma	Delta	Omicron
miR-148a-3p (94)	miR-148a-3p (94)	miR-148a-3p (94)	miR-148a-3p (94)	miR-148a-3p (94)	miR-148a-3p (94)
miR-148b-3p (94)	miR-148b-3p (94)	miR-148b-3p (94)	miR-148b-3p (94)	miR-148b-3p (94)	miR-148b-3p (94)
miR-152-3p (94)	miR-152-3p (94)	miR-152-3p (94)	miR-152-3p (94)	miR-152-3p (94)	miR-152-3p (94)
miR-4502 (88)	miR-4502 (88)	miR-4502 (88)	miR-4502 (88)	miR-4502 (88)	miR-4502 (88)
miR-5192 (88)	miR-5192 (88)	miR-5192 (88)	miR-5192 (88)	miR-5192 (88)	miR-5192 (88)
ORF7b					
Wuhan	Alpha	Beta	Gamma	Delta	Omicron
miR-589-5p (94)	miR-589-5p (94)	miR-589-5p (94)	miR-589-5p (94)	miR-589-5p (94)	miR-589-5p (94)
miR-6835-3p (91)	miR-6835-3p (91)	miR-6835-3p (91)	miR-6835-3p (91)	miR-6835-3p (91)	miR-6835-3p (91)
Nucleocapsid					
Wuhan	Alpha	Beta	Gamma	Delta	Omicron
miR-4684-3p (86)	miR-4684-3p (86)	miR-4684-3p (86)	miR-4684-3p (86)	miR-4684-3p (86)	miR-4684-3p (86)
miR-5682 (92)	miR-5682 (92)	miR-5682 (92)	miR-5682 (92)	miR-5682 (92)	miR-5682 (92)
miR-615-5p (79)	miR-615-5p (89)	miR-615-5p (79)	miR-615-5p (79)	miR-615-5p (79)	miR-615-5p (80)
miR-675-3p (87)	miR-675-3p (87)	miR-675-3p (87)	miR-675-3p (87)	miR-675-3p (87)	miR-675-3p (87)
3'UTR					
Wuhan	Alpha	Beta	Gamma	Delta	Omicron
miR-3941 (84)	miR-3941 (85)	miR-3941 (85)	miR-3941 (85)	miR-3941 (85)	miR-3941 (80)
